# All-optical observation on activity-dependent nanoscale dynamics of myelinated axons

**DOI:** 10.1117/1.NPh.10.1.015003

**Published:** 2023-01-23

**Authors:** Junhwan Kwon, Sungho Lee, Yongjae Jo, Myunghwan Choi

**Affiliations:** a Sungkyunkwan University, Department of Biomedical Engineering, Suwon, Republic of Korea; b Institute for Basic Science, Center for Neuroscience Imaging Research, Suwon, Republic of Korea; c Seoul National University, School of Biological Sciences, Seoul, Republic of Korea; d Seoul National University, The Institute of Molecular Biology and Genetics, Seoul, Republic of Korea

**Keywords:** spectral interferometry, optogenetics, nanoscale dynamics, myelinated axon, neuron

## Abstract

**Significance:**

In the mammalian brain, rapid conduction of neural information is supported by the myelin, the functional efficacy of which shows steep dependence on its nanoscale cytoarchitecture. Although previous *in vitro* studies have suggested that neural activity accompanies nanometer-scale cellular deformations, whether neural activity can dynamically remodel the myelinated axon has remained unexplored due to the technical challenge in observing its nanostructural dynamics in living tissues.

**Aim:**

We aim to observe activity-dependent nanostructural dynamics of myelinated axons in a living brain tissue.

**Approach:**

We introduced a novel all-optical approach combining a nanoscale dynamic readout based on spectral interferometry and optogenetic control of neural excitation in an acute brain slice preparation.

**Results:**

In response to optogenetically evoked neuronal burst firing, the myelinated axons exhibited progressive and reversible spectral redshifts, corresponding to the transient swelling at a subnanometer scale. We further revealed that the activity-dependent nanostructural dynamics was localized to the paranode.

**Conclusions:**

Our all-optical studies substantiate that myelinated axon exhibits activity-dependent nanoscale swelling, which potentially serves to dynamically tune the transmission speed of neural information.

## 1 Introduction

As neurons function by millisecond-scale ion flux across the cell membrane, neural activity has long been thought to accompany measurable morphological changes.^1^^–^^3^ Since the late 1970s, several groups have reported nanometer-scale swelling in the giant axons of invertebrate species (crayfish and squid) by Michelson interferometry^4^ and mechanoelectrical measurement.^5^ Later studies in mammalian cultured neurons by dark-field microscopy and full-field interferometry revealed the subnanometer-scale morphological dynamics dependent on electrical potential across the neuronal cell membrane.^4^^–^^8^

In contrast to the invertebrate models, the extent to which neural activity dynamically remodels the axons in living vertebrate brains has remained an enigma as these are orders-of-magnitude smaller and often ensheathed by the insulating layer of myelin. Myelin is a highly compacted subcellular structure of the oligodendrocyte, composed of multilayered lipid membranes and intervening aqueous mediums. This highly organized thin-film cytoarchitecture supports rapid and energy-efficient conduction of neural information in a small form factor, enabling formation of the highly integrated circuits of the mammalian brains.^9^^,^^10^ Considering that the function of myelin has a steep dependence on its thin-film structure, the structural dynamics of myelinated axons, even at the subnanometer scale, can have a critical impact on neural circuit functions.^11^

Investigating the nanostructural dynamics of myelinated axons in living mammalian brains is technically challenging. The current gold standard for nanoscale imaging of myelinated axons is electron microscopy, which is hardly applicable to living biological samples due to invasive sample preparation.^12^^–^^14^ Super-resolution techniques are a promising alternative for living biological samples, but subnanometer-scale precision has yet to be attained in the mammalian axons due to high optical aberrations of the lipid-rich myelin layers.^15^^–^^17^ Thus most studies have focused on long-term dynamics of relatively large morphological changes involving cell proliferation and differentiation.^11^^–^^13^^,^^18^

Several years ago, we developed a spectral interferometric technique, named SpeRe, that offers the nanoscale readout of the thin-film cytoarchitecture of the myelinated axons *in vivo*.^19^ Here we improved the SpeRe to have subnanometer spatial precision as well as subsecond temporal resolution and further combined it with optogenetic manipulation to unveil the neural activity-dependent nanostructural dynamics of myelinated axons in a living brain tissue. Our all-optical approach revealed for the first time that myelinated axons in a living mammalian brain exhibit subnanometer-scale swelling in response to neuronal burst firing and that the swelling dynamics is cumulative and reversible in the second scale.

## 2 Materials and Methods

### 2.1 Tapered Glass Fiber Sample

The tapered glass fibers were obtained by thermally drawing a glass rod (1.5-mm in diameter) using a micropipette puller (P-1000, Sutter Instrument). To mimic the typical axon diameters (1 to 5  μm), the optimal parameters for the micropipette puller were obtained by trial and error (ramp = 500 temperature = 550, pull = 40, velocity = 20, and pressure = 500). The tapered fiber was glued on a plain slide glass using an acrylic adhesive (401, Loctite) ensuring that the fiber was parallel to the slide glass (i.e., orthogonal to the optical axis of the objective lens). To firmly hold the fiber, a silicone-based sealant (Kwik-Cast, World Precision Instruments) was introduced around the fiber tip, exposing only the fiber tip in the air. The sample was imaged by a polarization microscope (SP8, Leica), and the diameter along the fiber was measured by applying the “distance map” module in ImageJ.

### 2.2 Artificial Cerebrospinal Fluid

Two types of artificial cerebrospinal fluid (aCSF) solutions were prepared; one is for surgery and the other is for recording. The surgical aCSF was composed of (in mM) 92 NMDG, 2.5 KCl, $1.25\;{\mathrm{NaH}}_{2}{\mathrm{PO}}_{4}$, $30\;\mathrm{NaH}{\mathrm{CO}}_{3}$, 20 HEPES, 25 glucose, 2 thiourea, 5 Na-ascorbate, 3 Na-pyruvate, $0.5\;{\mathrm{CaCl}}_{2}{\cdot 2H}_{2}O$, and $10\;{\mathrm{MgSO}}_{4}\cdot 7H_{2}O$. The recording aCSF was composed of (in mM) 124 NaCl, 2.5 KCl, $1.2\;{\mathrm{NaH}}_{2}{\mathrm{PO}}_{4}$, $24\;{\mathrm{NaHCO}}_{3}$, 12.5 glucose, $2\;{\mathrm{CaCl}}_{2}{\cdot 2H}_{2}O$, and $2\;{\mathrm{MgSO}}_{4}{\cdot 7H}_{2}O$ (Sigma Aldrich). Both solutions were titrated to have pH 7.3 to 7.4 and 300 to 310 mOsm while aerated with 95% $O_{2}$ and 5% ${\mathrm{CO}}_{2}$.

### 2.3 Mouse Preparation

All mice were housed with littermates in groups of two to five in a reverse day/night cycle and given ad libitum access to food and water. All animal experiments were performed in compliance with institutional guidelines and approved by the subcommittee on research animal care at Sungkyunkwan University and Seoul National University. Male or female C57BL6J wild-type mice aged 4 weeks (Jackson Laboratory) were used for virus-mediated transduction of a genetically encoded calcium indicator (GCaMP6s) and/or an optogenetic protein (ChrimsonR-tdT). The mouse was anesthetized by inhaling 4% isoflurane (Hanapharm) in an induction chamber and was subsequently maintained with 1% to 1.5% isoflurane during surgery. The mouse skull was affixed on a custom-made stereotaxic frame and the body temperature was maintained at 37°C using a homeothermic blanket (TC-1000, CWE) and a thermistor probe (YSI-451, CWE). After removing the scalp, the hole was made on the center of the somatosensory area at a diameter of ∼1  mm. The 700-nL of a solution containing AAV9- hSyn-GCaMP6s and/or AAV5-hSyn-Chrimson-tdTomato ($∼5\times 10^{11}\;\mathrm{GC}\cdot {\mathrm{ml}}^{-1}$ each in the recording aCSF) was slowly infused to cortical layers 3 and 4. After 3 weeks, the mice were used for the acute slice experiments.

### 2.4 Brain Slice Preparation

Mice were decapitated under deep anesthesia by inhaling 3% isoflurane in $O_{2}$. The mouse brain was harvested and sliced using a vibratome (thickness=300  μm; VT1200S, Leica). During the slicing procedure, the immersion solution was the surgical aCSF solution kept at 4°C. Subsequently, the brain slices were incubated in the surgical aCSF solution at 35°C for 20 min and were immersed in the recording aCSF solution at room temperature (22°C to 24°C) with continuous aeration of 95% $O_{2}$ and 5% ${\mathrm{CO}}_{2}$ for 30 min. The brain slices were mounted on an imaging chamber using a tissue anchor (SHD-41/10, Warner instruments). For the neuronal inhibition study, tetrodotoxin (TTX) was added to the recording aCSF at 10  μM. Damaged neurons were excluded based on their morphology if blebbing or a ruptured membrane was observed in a DIC image. Functional viability was subsequently confirmed using calcium responsiveness under optogenetic stimuli.

### 2.5 Optic Setup

**Fig. 1:**
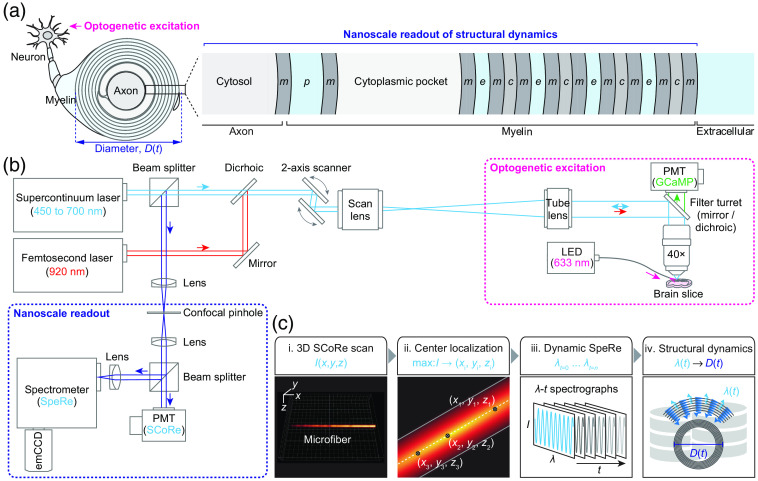
All-optical approach for observing activity-dependent nanostructural dynamics of living myelinated axons. (a) A cross-sectional view on the nanoscale cytoarchitecture of a myelinated axon at the paranodal region. The thin-film layers of the myelinated axons are shown in the magnified view. m, membrane; p, periaxonal space; c, cytosolic layer of the myelin; e, extracellular region; D, diameter; and t, time. (b) The optic setup incorporating SCoRe, spectral reflectometry (SpeRe), two-photon fluorescence imaging, and optogenetic excitation. The supercontinuum laser serves as a light source for SpeRe and SCoRe, and the femtosecond laser provides two-photon excitation for recording GCaMP-mediated neuronal calcium activity. The auxiliary fiber-coupled LED at 633 nm was used for triggering ChrimsonR-mediated excitatory optogenetics. (c) A pipeline for the nanoscale readout of structural dynamics of a myelinated axon. (i) 3D SCoRe scan to acquire a volumetric reflectance image of myelinated axons. (ii) Localization of the geometric centers based on the maximal reflectance intensity. (iii) Time-lapse acquisition of the reflectance spectrographs at the geometric center. (iv) Decoding of the structural dynamics from the acquired spectrographs. I, intensity and λ, wavelength.

Our customized optic system shown in Fig. 1(b) was designed to incorporate the following three modalities: (i) two-photon fluorescence imaging for recording neuronal activity; (ii) spectral reflectance spectroscopy for nanostructural readout of myelinated axons; and (iii) optogenetics for manipulating neural activity. The system was constructed based on an upright galvanometer-based laser scanning microscope (Ultima IntraVital, Bruker), coupled to a Ti-Sapphire femtosecond laser (for two-photon fluorescence imaging; Chameleon Ultra II, Coherent) and a supercontinuum white-light laser (for SpeRe and SCoRe; EXB-6, NKT photonics). The femtosecond laser was tuned to 920 nm for exciting GCaMP6s and was attenuated to 10 to 20 mW at the objective back aperture. The supercontinuum laser was attenuated to ∼0.4  mW at the objective back aperture using a neutral density filter and bandpass filtered to 450 to 700 nm. An apochromatic water-immersion objective lens (25×, 0.95 NA, Leica) was used for both two-photon fluorescence and SpeRe/SCoRe readouts. For two-photon fluorescence imaging, a GaAsP photomultiplier tube placed at the nondescanned path was used along with a bandpass filter at 500 to 550 nm. For the SCoRe imaging, a silicon photomultiplier tube placed at the descanned path was used. For SpeRe measurements, an array spectrometer (SR303i and Newton, Andor) was introduced at the descanned path. For spectroscopy, the grating of $600\;\text{lines}\cdot {\mathrm{mm}}^{-1}$ was adjusted to accept a spectral window of 550 to 650 nm, where the input white light exhibited near uniform intensity profile over the spectral window. The slit size was set to 10  μm, providing a spectral resolution ∼0.13  nm with enough signal-to-noise ratio at the acquisition speed of 20 Hz. For optogenetics, a 633-nm diode laser (MRL-III-633, CNI laser) was coupled to a multimode fiber (400  μm core, 0.39 NA; M119L02, Thorlabs), which was mounted on a motorized three-axis micromanipulator (MP-285, Sutter Instrument). Optical irradiance for optogenetic stimulation was set to $10\;\mathrm{mW}\cdot {\mathrm{mm}}^{-2}$ at the tissue surface, which was delivered at 100 Hz with 50% duty cycle for the duration of 0.5 to 2 s.

### 2.6 Data Analysis

For SpeRe, the time-series reflectance spectra were filtered in the spectral and time domains in MATLAB. To reduce artifactual jittering noise, the low-pass filter at a cutoff frequency of $0.65\;{\mathrm{nm}}^{-1}$ was applied in the wavelength domain by applying the “low-pass” function, and the smoothing filter with the bin of 0.5 s was applied in the temporal domain using the “smooth data” function. From the filtered spectral data, the relative phase shift over time from the baseline was retrieved based on the least-squares method. Occasionally, unpredictable motion artifacts (e.g., instability of media perfusion) interfered reliable quantification of the phase shift. Thus we excluded the data if it displayed at least one of the following indications of excessive motion artifact: change in reflectance intensity >10% or the phase drifted over 3 nm. For calcium imaging data, we quantified relative change in fluorescence normalized by the baseline fluorescent intensity (ΔF/F).

### 2.7 Statistical Analysis

GraphPad Prism was used for statistical analysis. Group comparisons were conduction using unpaired t-tests (parametric). The data are presented as mean ± standard error. We considered a p-value of <0.05 to be statistically significant.

## 3 Results

### 3.1 System for All-Optical Investigation

To observe the morphological dynamics of functionally active myelinated axons at the nanoscale, we introduced a all-optical neurophysiology approach [Figs. 1(a) and 1(b)]. For manipulating neural excitation with minimal mechanical perturbation, we introduced red-shifted excitatory optogenetic protein (ChrimsonR) into cortical excitatory neurons. Neuronal excitation was timely triggered by an epi-illuminated fiber-coupled light-emitting diode (LED) at 633 nm on an acute brain slice and was confirmed by GCaMP-mediated functional calcium imaging on the targeted neurons by two-photon microscopy.^20^ For recording nanostructural dynamics of myelinated axons, we incorporated spectral reflectometry (SpeRe) that captures broadband reflectance spectra at the geometric center of the axons and decodes the physical size of the multilayered thin films (i.e., diameter of the myelinated axons) by decoding the spectrums. To assist with pinpointing the geometric centers, we additionally introduced spectral confocal reflectance imaging (SCoRe),^21^ which provides a volumetric image of reflected light corresponding to the centerlines of myelinated axons.

The overall procedure for nanostructural readout begins with acquisition of a volumetric reflectance image from a fresh brain slice by SCoRe [Fig. 1(c)]. From the volumetric SCoRe image, the position of maximum intensity for each cross section is localized, resulting in the geometric centerline along the fibrous structure. The broadband reflectance spectrum is subsequently acquired over time at the center positions, and the structural dynamics is decoded from the acquired spectra (SpeRe).

For decoding of nanostructural dynamics, we performed numerical optic simulation on the myelinated axons and obtained quantitative relationship between the reflectance spectrum and the nanoscale cytoarchitecture. Briefly, the interaction of light waves at the subcellular layers of myelinated axons was described by the thin-film matrix theory, and the distribution of light waves at the focus was formulated by the vector diffraction theory. As the morphological dynamics were observed exclusively at the paranode of myelinated axons in our following experiments, we set the simulation parameters including physical size and refractive index for each subcellular layer based on the paranodal region (Table S1 in the Supplementary Material).

The resulting simulation database showed that the swelling of myelinated axons (ΔD>0) leads to negative phase-shift in the wavenumber domain (Δv<0) with linear relationship (Fig. S1 in the Supplementary Material). Thus we decided to use the relative phase shift (Δν) as a reliable metric to quantify the dynamic swelling of myelinated axons (i.e., phase detection method^22^^–^^24^). To detect subnanometer-scale morphological dynamics, we used a grating of $600\;\text{lines}\cdot {\mathrm{mm}}^{-1}$ and a slit size of 10  μm, resulting in a spectral resolution of ∼0.13  nm.

### 3.2 Validation of Subnanometer-Scale Readout Precision

**Fig. 2:**
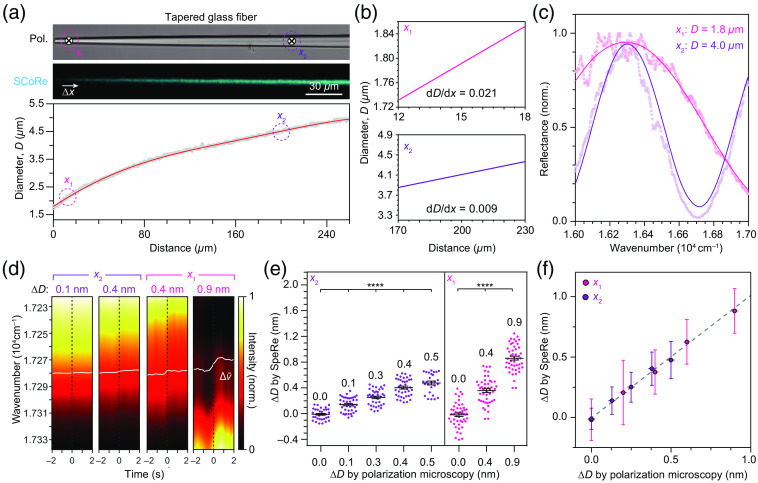
Validation on nanometer-scale readout precision of SpeRe. (a) A tapered glass fiber sample for quantifying the nanoscale readout precision. The tapered fiber is imaged by polarization microscopy (Pol.) and SCoRe. The gradually varying diameter (D) along the fiber was acquired from the polarization microscopic image. (b) The spatial derivatives of the diameter (dD/dx) at the representative positions ($x_{1}$ and $x_{2}$) along the tapered glass fiber. (c) Reflectance spectrographs at the positions $x_{1}$ and $x_{2}$. The acquired spectrographs (dots) were overlaid with best-fit simulated spectra (curved lines). (d) Representative time-lapse reflectance spectrographs measured at the indicated positions ($x_{1}$ for ΔD=0.4 and 0.9 nm; $x_{2}$ for ΔD=0.1 and 0.4 nm) with step shifts in galvo-mirror position at 0 s. The step change in position was introduced by shifting the focal point along the centerline of the fiber. The white curves indicate the relative spectral shift. (e), (f) Change in diameters (ΔD) estimated by SpeRe with the corresponding estimates by polarization microscopy. *p<0.0001 (unpaired t-test). The error bars indicate standard deviations.

SpeRe was previously applied for measuring nanoscale morphological dynamics, such as osmotic swelling of myelinated axons or protein adhesion to a functionalized microsphere.^19^^,^^25^ To further verify the subnanometer-scale precision of our SpeRe readout, we used a finely tapered glass fiber prepared by thermally drawing a glass rod using a micropipette puller (Fig. 2). In a tapered fiber, the diameter (D) is a smooth function of its longitudinal position (x); therefore, we can simply mimic the nanoscale change in diameter (ΔD) by shifting the position (Δx) using a galvanometric scanner. We first estimated the diameter along the fiber using an image acquired by polarization microscopy and validated by SpeRe [Figs. 2(a) to 2(c)]. We then selected two points, $x_{1}$ and $x_{2}$, which showed physiological axon diameters (0.3 to 5  μm). By the linear regression near the selected points, we acquired the slope (dD/dx), which provided the multiplicative factor for converting the step shift in position (Δx) to the change in diameter (ΔD). For example, to introduce an increase in diameter by 0.5 nm at $x_{2}$, we shifted the position along the centerline by +55  nm, corresponding to ΔD (0.5 nm) divided by dD/dx (0.009).

In a tapered fiber, we obtained broadband reflectance spectra over time on the two selected positions at a sampling speed of 20 Hz. To convert the spectral shift (Δv) to the change in physical diameter (ΔD), we applied the linear relationship derived from our numerical simulation for each position (Figs. S2a–S2c in the Supplementary Material). The in-position stability of SpeRe acquisition during the initial 2 s was ∼±0.2  nm at $x_{1}$ and ±0.1  nm at $x_{2}$ in standard deviation (Figs. S2d–S2f in the Supplementary Material). The step shifts in the scan position (Δx) with variable distances were introduced at 2 s after the acquisition [Figs. 2(d)–2(f)]. By the step shift in the scan position, we observed the reliable negative spectral shift in wavenumber domain (Δv<0), corresponding to the swelling. The estimated changes in diameter by SpeRe were precisely matched with the inputs introduced by the shift in position [Figs. 2(e)–2(f)]. These results suggest that our dynamic SpeRe readout provides subnanometer-scale precision.

### 3.3 Activity-Dependent Nanostructural Dynamics of Myelinated Axons

**Fig. 3:**
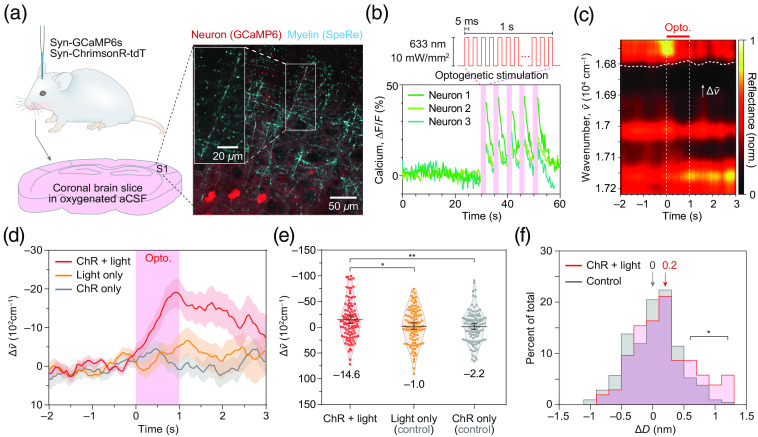
All-optical observation of activity-dependent subnanometer-scale swelling of myelinated axons. (a) Sample preparation. The neurons in the somatosensory cortex (S1) were transfected with a fluorescent calcium indicator (GCaMP6s) and an optogenetic actuator (ChrimsonR). A representative fluorescence/reflectance image from the brain slice is shown on the right. (b) Optogenetic excitation. Neurons transfected with ChrimsonR was excited by illuminating pulsed light (wavelength: 633 nm, irradiance: $10\;\mathrm{mW}\cdot {\mathrm{mm}}^{-2}$, frequency: 100 Hz, and pulse width: 5 ms). Calcium traces in soma from three neurons in (a) are shown. The shaded areas in pink indicate the duration of optogenetic stimulation. (c) A representative phase shifts ($\Delta \overset{˜}{v}$) induced by the optogenetic neural activity. The red bar indicates the duration of optogenetic stimulus (1 s). The white dashed line indicates the relative phase shift from the baseline (−1.0 to −0.5  s). (d) The group averaged phase shifts ($\Delta \overset{˜}{v}$) for each experimental group. The red curve (ChR + light group, n=104 axons in 5 mice) indicates the ChrimsonR-expressing neurons with light stimulus, the yellow curve (“light only” group, n=105 axons in 5 mice) indicates the wild-type neurons receiving photostimulation, and the gray curve (ChR only group, n=105 axons in 5 mice) indicates the ChrimsonR-expressing neurons without light stimulus. Note that only the ChR + light group exhibited a distinguishable negative shift in wavenumber. (e) Statistical group comparison of phase shifts ($\Delta \overset{˜}{v}$) in (d). The phase shifts were quantified by averaging the relative phase shifts during 0.5 to 1 s. *p<0.05 (unpaired t-test). **p<0.01 (unpaired t-test). (f) Histograms of activity-dependent changes in diameters of myelinated axons (ΔD). The positive value of ΔD corresponds to swelling.

For optical manipulation and recording of neural excitation, we microinjected two types of adenoassociated viruses encoding a red-shifted opsin (AAV9-hSyn-ChrimsonR-tdTomato) and a fluorescent calcium indicator (AAV9-hSyn-GCaMP6s) at the somatosensory cortex of live mice and prepared fresh brain slices in oxygenized medium on the experiment day^26^^,^^27^ [Fig. 3(a)].

By combining two-photon fluorescence and confocal reflectance imaging of the brain slice, we visualized transfected neurons, as well as their myelinated axons. By illuminating 633-nm light pulses following the high frequency stimulation protocol, we observed reliable functional calcium activity in the most neuronal soma expressing both ChrimsonR and GCaMP6s^10^^,^^13^ [Fig. 3(b) and Fig. S3 in the Supplementary Material].

To address whether neuronal excitation leads to nanostructural dynamics of myelinated axons, we randomly sampled up to 10 myelinated axons for each brain slice and recorded the nanoscale dynamics by SpeRe for 5 s with light stimuli [“ChR + light” group, n=104 axons in 5 mice; Figs. 3(c) and 3(d)]. We specifically targeted the *en passant* axons in the periphery of the viral injection site due to their inherent sparsity. As negative control groups, we included brain slices without light stimuli (“ChR only” group, n=105 axons in 5 mice) and brain slices without introducing the optogenetic actuator (“light only” group, n=105 axons in 5 mice). The SpeRe readout was performed near either end of the myelin sheaths, corresponding to the paranode, where axomyelinic communication is known to be active.^28^^–^^30^ Apparently, only the group with functional optogenetic excitation (ChR + light group) showed statistically significant spectral shift by $∼2000\;{\mathrm{cm}}^{-1}$ on average [unpaired t-test: p<0.05; Fig. 3(e)]. Intriguingly, the spectral shift was cumulatively increased during the 1-s period of optogenetic excitation and slowly recovered in several seconds, indicating that the activity-dependent morphological dynamics does not follow the neuronal membrane potential having millisecond-scale rise and fall kinetics. Moreover, pharmacological inhibition of action potential generation by TTX significantly, but only partially, attenuated the swelling, suggesting that the generation of action potentials is not necessary for inducing the swelling (Fig. S4 in the Supplementary Material).

To convert the spectral shift (Δv) to the change in physical diameter (ΔD), we applied the linear relationship derived from our numerical simulation (Fig. S1 in the Supplementary Material). As the nanostructural parameters for individual myelinated axons that we sampled were not attainable, we applied the representative structural parameters in Table S1 in the Supplementary Material obtained from previous electron micrographs and estimated from our optical images (Fig. S3 in the Supplementary Material).^19^^,^^31^ Although this approach compromised the precision of estimation on individual axons, we were able to gain information on the population distribution. Although statistically significant, the group-averaged spectral shift of $∼2000\;{\mathrm{cm}}^{-1}$ corresponds to ∼0.2  nm, which was at least several folds smaller than previously reported axonal swelling observed in cell culture and invertebrate systems [Fig. 3(e)]. This discrepancy can be explained by our experimental design, which randomly samples myelinated axons in brain slices. Conceivably, a significant portion of long-projecting myelinated axons is expected to be severed during the tissue slicing procedure and contains insufficient optogenetic proteins due to the stochastic nature of viral transfection. Consequently, the large portion of the samples even in the ChR + light group might have been nonresponsive to optogenetic stimuli. Indeed, the histograms of change in diameters for the experimental and the negative control groups were largely similar with statistically significant differences observed only at the swelling >∼0.5  nm [Fig. 3(f)].

**Fig. 4:**
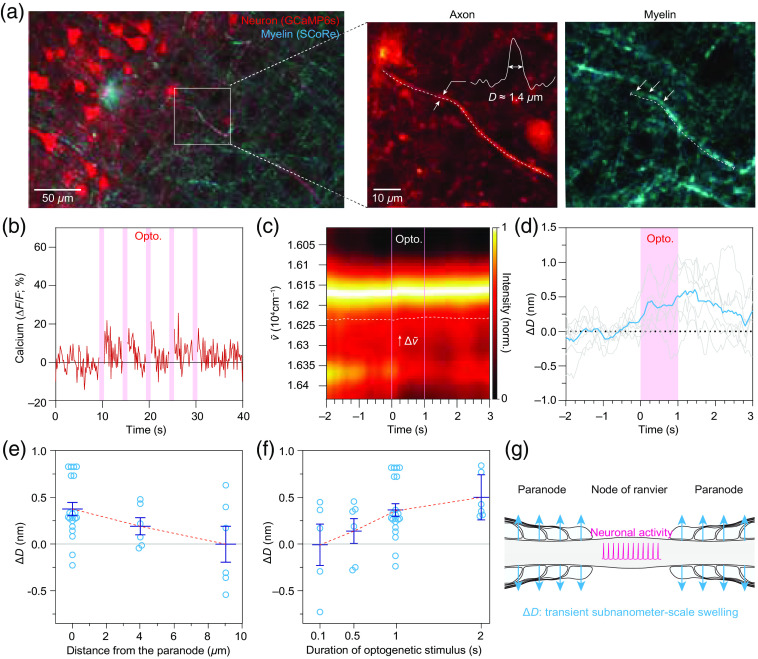
Correlative characterization of subnanometer-scale swelling in an optogenetically active axon. (a) Representative images of myelinated axons. The neurons are imaged by two-photon fluorescence of GCaMP6s (red), and the myelin is visualized by SCoRe (cyan). The magnified view of the myelinated axon of interest is shown on the left. The axon diameter (D) was estimated to by full-width-half-maximum of the transverse intensity profile (D≈1.4  μm). The dashed lines indicate the axon and the myelin sheath. (b) Axonal calcium activity triggered by optogenetic excitation of ChrimsonR. The shaded area in magenta indicates the duration of optogenetic excitation (633 nm, $10\;\mathrm{mW}\cdot {\mathrm{mm}}^{-2}$). (c) Observation of time-dependent change in reflectance spectrum at the optogenetically active myelinated axon. The dashed lines indicate the duration of optogenetic stimulation. Note the progressive spectral redshift ($\Delta \overset{˜}{v}$) during the optogenetic stimulation. (d) Time-dependent changes in the diameter of myelinated axons (ΔD) in response to optogenetic stimulation (shaded region in magenta). The cyan curve indicates the averaged trace of seven repeated trials (individual traces are shown in grey). (e) The swelling is localized to the paranode. (f) Dependency of ΔD on the duration of optogenetic stimuli at the paranode. (g) The schematic diagram of the activity-dependent nanoscale dynamics of the myelinated axons.

We thus questioned if we could observe reliable nanostructural dynamics in the myelinated axons that are morphologically intact and functionally active (Fig. 4). By two-photon imaging of neuronal morphology and functional calcium activity with optogenetic stimuli, we identified the three morphologically intact and functionally active myelinated axons, which were connected to the intact neuronal soma [Figs. 4(a) and 4(b)]. As expected, the three myelinated axons repeatedly exhibited a progressive increase in their diameters by 0.3 to 1 nm in response to optogenetic stimuli [Figs. 4(c) and 4(d) and Fig. S5 in the Supplementary Material). We further observed that the morphological dynamics was localized to the paranode [Fig. 4(e)] and that the degree of swelling was positively correlated with the duration of optogenetic stimuli [Fig. 4(f)].

## 4 Discussion

By combining the nanoscale readout based on spectral interferometry (SpeRe) and optogenetic manipulation of neural excitation, we report the first experimental evidence that myelinated axons exhibit activity-dependent subnanometer-scale morphological dynamics of ∼0.5  nm. We further reveal that the nanostructural change displays slow kinetics at the second scale and localized to the paranode. As conduction efficacy of neural information has steep dependence on the subcellular structure of the paranode, we expect that its nanostructural dynamics can serve as a regulatory mode of controlling conduction speed in the mammalian brains.

The functional consequence of the morphological remodeling at the paranode can be inferred by the periaxonal nanocircuit model of the myelinated axons.^10^ According to this model, the periaxonal space is electrically conductive and the paranodes are only partially sealed, resulting in leaky propagation of neural information. Thus the swelling of the paranode can attenuate the leakage and accelerate the conduction speed. By the theoretical double cable model, 1-nm swelling at the paranode leads to ∼1.3% acceleration in conduction speed. Accordingly, the observed swelling of ∼0.5  nm is expected to increase the conduction speed by ∼0.7%. Consistently, Yamazaki et al.^29^^,^^32^ reported a short-term increase in conduction speed by directly depolarizing the myelinating oligodendrocyte. In addition, the paranodal remodeling may also involve analog modulation of action potential waveform,^33^ which potentially affects synaptic coupling^34^ and network synchrony.^35^ Furthermore, fields and Ni reported that unmyelinated axons swell in response to action potential firing, leading to nonvesicular release of adenosine triphosphate from axons through volume-activated anion channels,^36^^,^^37^ suggesting potential transmitter-mediated interaction between axons and nearby glia. The physiological relevance of the paranodal remodeling on neural circuit function requires further investigation.

Our results reveal that the paranodal swelling occurs cumulatively and reversibly at the second scale, suggesting that the neuronal membrane potential having a millisecond-scale rise and fall kinetics is not the direct source of the swelling [Figs. 3(d) and 4(d)]. In addition, pharmacologic inhibition of voltage-gated sodium channel by TTX only partially attenuated the paranodal swelling (Fig. S4 in the Supplementary Material), suggesting that the generation of action potential is not necessary for inducing the swelling. Considering that ChrimsonR is a light-gated channel permeable to both sodium and calcium ions,^27^^,^^38^ we expect that the calcium influx may play a key role in the paranodal swelling. In agreement with our thought, we observed that calcium transient was evoked by ChrinsonR-mediated optogenetics even with TTX-mediated inhibition of the voltage-gated sodium channel. The partial decline in calcium activity was correlated with the attenuation of paranodal swelling, also supporting the causal role of calcium activity in the swelling. Further pharmacological inhibition of ion channel subtypes or use of ion-selective opsins will clarify the underlying molecular mechanism.

Although our results consistently suggest that the neuronal excitation leads to enlargement of myelinated axons at the paranode, which subcellular components are remodeled remains to be answered. Chéreau et al. reported that high-frequency stimulation of neurons leads to axonal swelling in the mouse brain.^15^ Trigo and Smith also showed micron-scale axonal swelling following prolonged electric stimulation of the peripheral nerves.^28^ As mature myelin sheaths do not typically exhibit axonal activity-dependent functional responses, we estimate that the axon at the node of Ranvier swells in response to ionic redistribution across the membrane and that the surrounding myelin is passively enlarged. Detailed multiparametric analysis based on the thin-film model with *a priori* structural information on each subcellular composition may provide a solution to this question. Alternatively, subnanometer-scale super-resolution imaging techniques (e.g., MINFLUX^39^ and SUSHI^40^) will help to reveal further details in activity-dependent structural dynamics.

## 5 Summary

As neural activity involves rapid ion flux across the cell membranes, researchers have long tried to detect the accompanying nanoscale morphological dynamics. However, measuring the activity-dependent nanostructural dynamics in the living mammalian brain has been an enigma due to the technical limitations. By combining excitatory optogenetics and *in situ* nanoscale metrology based on spectral interference, we demonstrate the first direct observation that the mammalian axons exhibit transient activity-dependent swelling at the subnanometer scale.

## Code, Data, and Materials Availability

All relevant data are available from the corresponding author upon request. A reporting summary for this article is available as a Supplementary Material file. The MATLAB scripts used for data analysis are available at an open-source repository (https://github.com/Neurophoton).
