# Supplementary material for: All-optical observation on activity-dependent nanoscale dynamics of myelinated axons
Source: Neurophotonics. 2023 Jan 23;10(1):015003. doi: 10.1117/1.NPh.10.1.015003 (PMC9868287; doi:10.1117/1.NPh.10.1.015003)
Supplement: Supplementary file 1 [file NPh_010_015003_SD001.pdf]

All-optical observation on activity-dependent nanoscale dynamics of myelinated axons

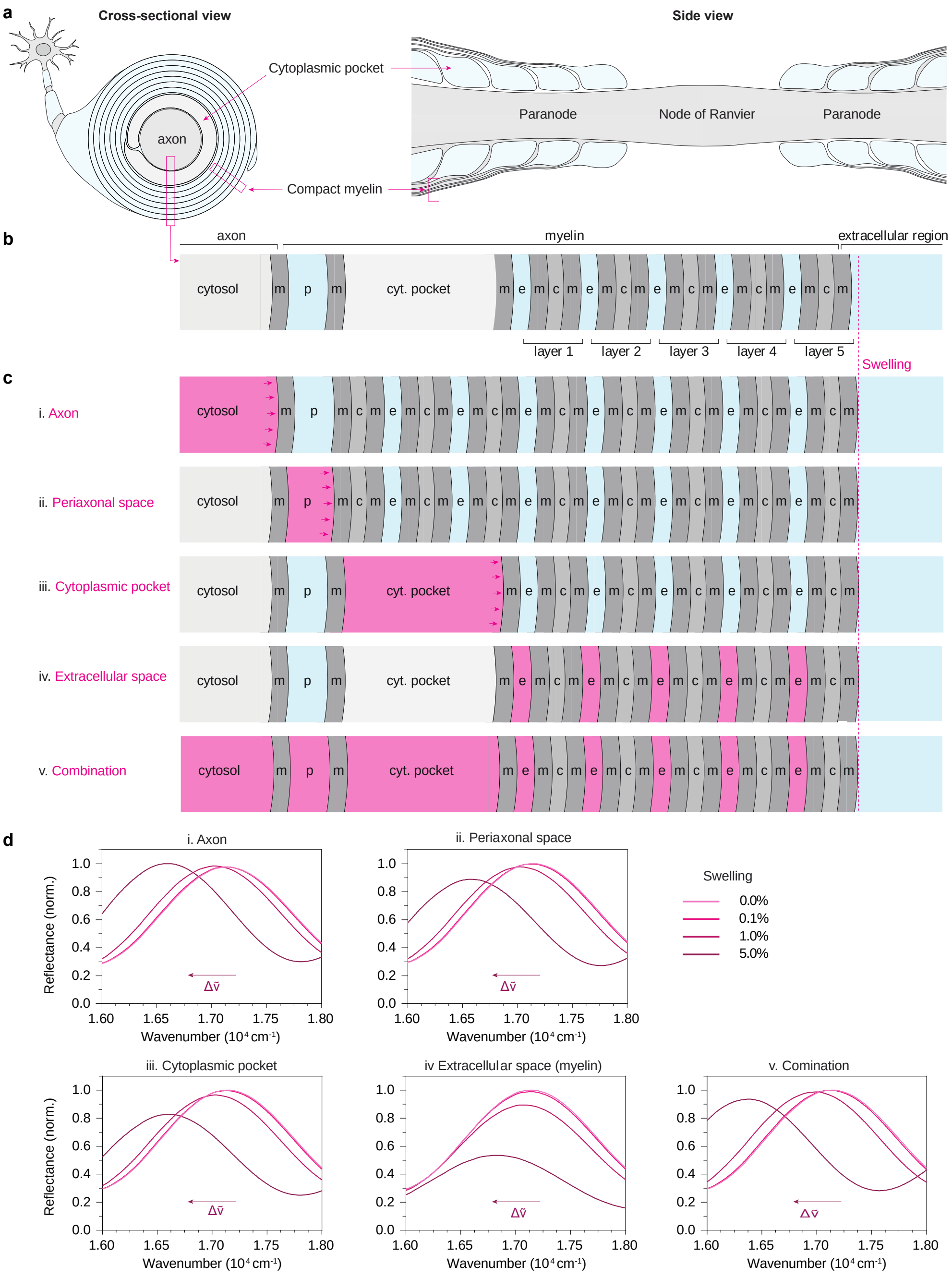

Fig. S1. The spectral shift represents nanoscale structural change. (a) Schematic illustrations of a neuron with a myelinated axon fiber. Left, a cross-sectional view of a myelinated axon at the paranodal region. Right, a side view of a myelinated axon at the node and paranodal regions. Note that the cytoplasmic pocket is enlarged at the paranodal

region. (b) A thin-film model for the paranodal region. (c) Potential swelling modes of myelinated axons. The affected subcellular regions are shown in magenta. i, the axon is swollen. ii, the periaxonal space is enlarged. iii, the cytosolic pockets are swollen. iv. the extracellular spaces of myelin is swollen. v. combination of the above. (d) Spectral shift in SpeRe for the 5 cases shown in (c). Note that for all the cases, the swelling in the cavity size leads to the spectral redshifts in the similar degree, suggesting that the spectral shift can serve as a reliable indicator for tracing the nanoscale swelling dynamics of myelinated axons.

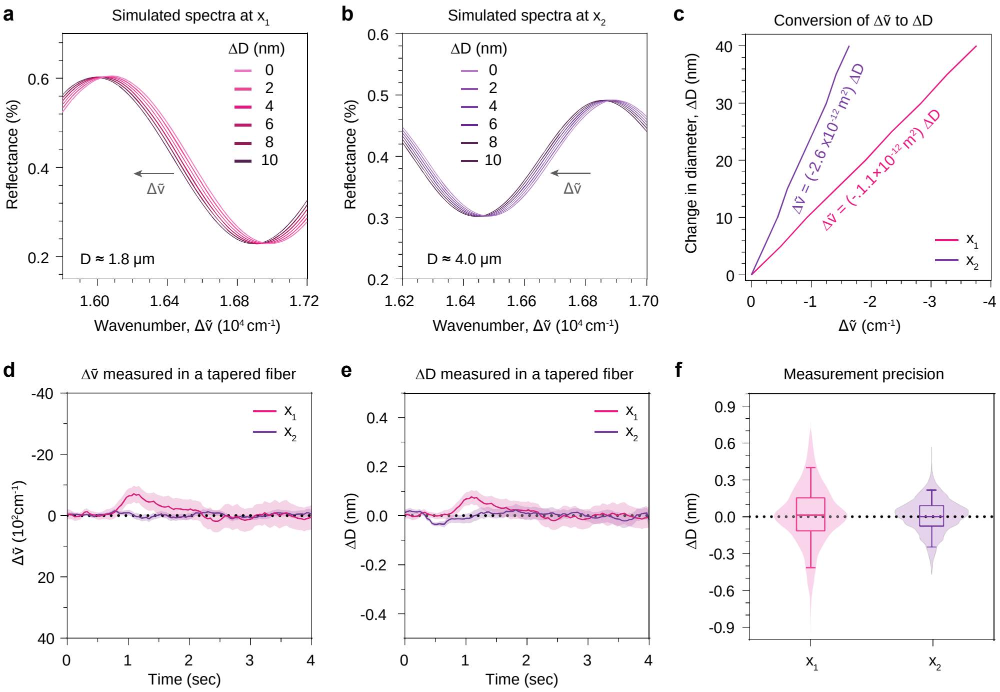

Fig. S2. Analysis pipeline for the stable SpeRe acquisition. (a,b) In a tapered glass fiber sample shown in Fig. 2a, numerical simulation produced reflectance spectrums while increasing the diameters with 2 nm increments for the two locations (base x1, 1.8 µm and x2, 4 µm). (c) the linear relationships between the spectral shifts (Δṽ) and the diameter increases (ΔD) acquired from (a,b). (d-f) the SpeRe acquisition was performed at a sample rate of 20 Hz for a period of 6 s. (d) Artifacts in spectral shift over time. The SpeRe acquisition (Δṽ, relative shift in wavenumber) was performed at the two locations on the tapered fiber indicated in Fig. 2a (x1 and x2). (e) Converting the spectral shift (Δṽ) to the change in diameter (ΔD). Using simulation database, the relationship between Δṽ and ΔD was deduced and applied. (f) Precision of the dynamic SpeRe acquisition. The stability in standard deviation at x1 was 0.2 nm and that at x2 was 0.1 nm.

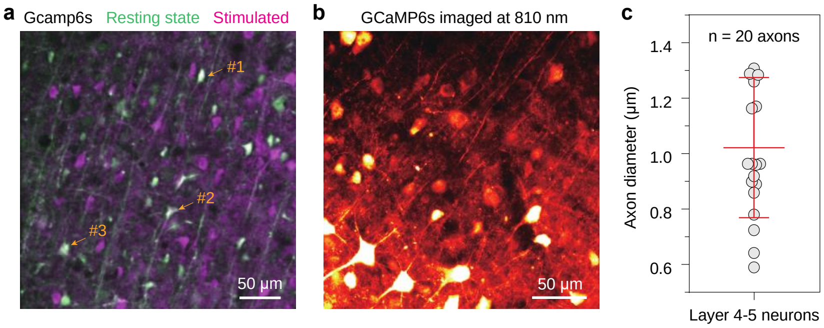

Fig. S3. Physical parameters of the myelinated axons. (a) A representative image of a mouse brain slice virally transfected with a genetically encoded calcium indicator and an optogenetic actuator. Resting state neurons (green) and activated neurons by optogenetic light (magenta) of GCaMP6s imaged by two-photon at the functional imaging wavelength (920 nm). (b) Anatomical image of neurons and their axon fibers. This pseudo-color image is acquired from two-photon fluorescence of GCaMP6s excited at the isosbestic wavelength (810 nm). (c) Quantified axon diameters from the image acquired in (b). Axon diameters originated from the layer 4-5 neurons in the somatosensory area were acquired by quantifying full-width-half-maximum of the transverse intensity profile (n = 20 axons). The error bar indicates standard deviation.

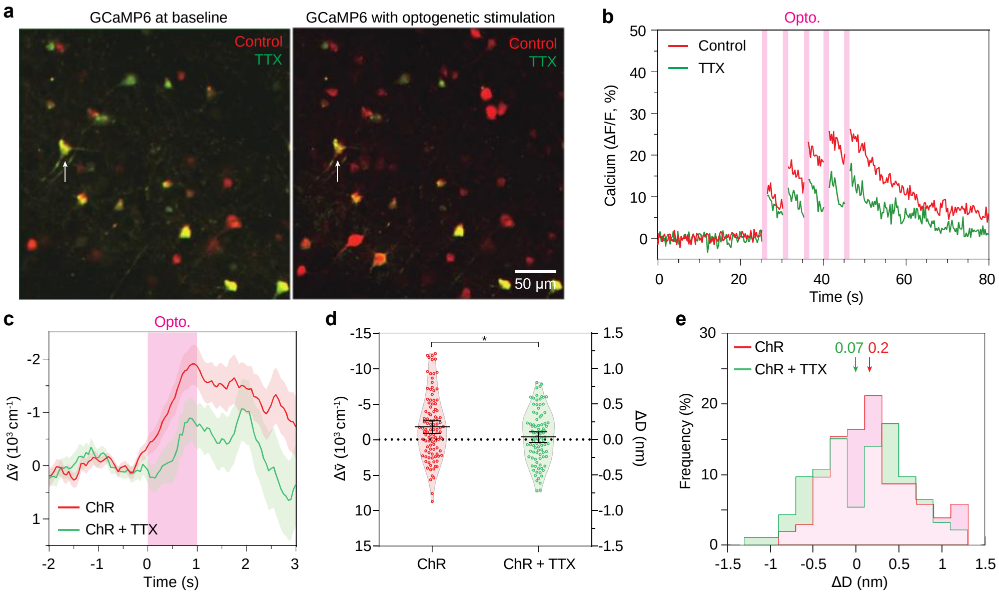

Fig. S4. Inhibition of voltage-gated sodium channel by tetrodotoxin (TTX) only partially inhibited the activity-dependent swelling of myelinated axons. A brain slice from a virally transfected with GCaMP6s and ChrimsonR-tdTomato was bathed with 0 or 10 μM TTX in aCSF. (a) Merged Gcamp6s image of neurons taken by two-photon imaging. Left, Resting state of Gcamp6s signal. Right, Activated neurons by optogenetic stimulation. Red, Neurons in aCSF with no TTX. Green, Neurons in aCSF with 10 μM TTX. Both images exhibited attenuated Gcamp6s signals by TTX. (b) T-series calcium changes. Calcium transient of neurons in TTX medium exhibited attenuated amplitude in ΔF/F. (c) Comparison of time-series phase shift. Pink shadow, period of optogenetic stimulation. Solid line, averaged trace for each group. Shadow, standard error of mean. (d) Maximum phase difference between mean of baseline (-1 to -0.5) and mean of stimulated period (0.5 to 1) in (c). (e) Comparison of distributions of maximum diameter changes estimated by SpeRe. TTX, n = 93 myelinated axons from 4 mice.

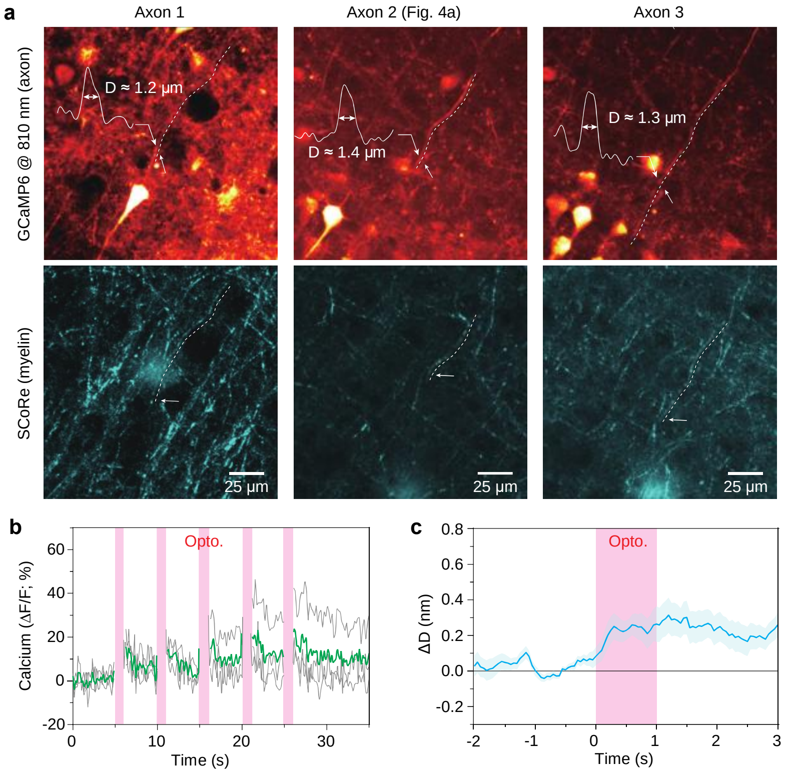

Fig. S5. Correlative observation on activity-dependent subnanometer-scale swelling of optogenetically active myelinated axons. (a) The representative cases of optogenetically active myelinated axons (n = 3 axons). Upper, axons imaged by two-photon fluorescence imaging at isosbestic wavelength (810 nm). Lower, Myelin sheath wrapping optogenetically active axons visualized SCoRe (cyan). The axon diameters were estimated to by full-width-half-maximum of transverse intensity profile. (b) All axons in (a) consistently exhibited rise in intracellular calcium by optogenetic stimulation (shaded in magentha). (c) Averaged traces of activity-dependent change in diameter (ΔD) measured by SpeRe. The shaded cyan area indicates the standard error of the mean.

Table S1. Optical parameter for numerical simulation

Subcellular structure	Size	Refractive index
Axon	1 µm	1.36
Membrane	5 nm	1.50
Cytoplasmic pocket	200 nm	1.35
Cytosol (compact myelin)	3 nm	1.47
Extracellular space (myelin)	5 nm	1.36
Periaxonal space	12 nm	1.35
# of myelin layers	5	-

The physical sizes and refractive indices of myelinated axons at the paranode used in the numerical simulation
